# Enhancing the scoping study methodology: a large, inter-professional team’s experience with Arksey and O’Malley’s framework

**DOI:** 10.1186/1471-2288-13-48

**Published:** 2013-03-23

**Authors:** Helena ML Daudt, Catherine van Mossel, Samantha J Scott

**Affiliations:** 1 Clinical Research, British Columbia Cancer Agency, Vancouver Island Centre, 2410 Lee Avenue, Victoria, BC V8R 6V5, Canada; 2 University of Victoria, 1223 Oxford St., Victoria, BC V8V 2V6, Canada; 3 Interior Health Authority, Royal Inland Hospital, 311 Columbia Street, Kamloops, BC V2C 2T1, Canada

**Keywords:** Scoping study, Methodology, Inter-professional team, Colorectal cancer, Information needs

## Abstract

**Background:**

Scoping studies are increasingly common for broadly searching the literature on a specific topic, yet researchers lack an agreed-upon definition of and framework for the methodology. In 2005, Arksey and O’Malley offered a methodological framework for conducting scoping studies. In their subsequent work, Levac et al. responded to Arksey and O’Malley’s call for advances to their framework. Our paper builds on this collective work to further enhance the methodology.

**Discussion:**

This paper begins with a background on what constitutes a scoping study, followed by a discussion about four primary subjects: (1) the types of questions for which Arksey and O’Malley’s framework is most appropriate, (2) a contribution to the discussion aimed at enhancing the six steps of Arskey and O’Malley’s framework, (3) the strengths and challenges of our experience working with Arksey and O’Malley’s framework as a large, inter-professional team, and (4) lessons learned. Our goal in this paper is to add to the discussion encouraged by Arksey and O’Malley to further enhance this methodology.

**Summary:**

Performing a scoping study using Arksey and O’Malley’s framework was a valuable process for our research team even if *how* it was useful was unexpected. Based on our experience, we recommend researchers be aware of their expectations for how Arksey and O’Malley’s framework might be useful in relation to their research question, and remain flexible to clarify concepts and to revise the research question as the team becomes familiar with the literature. Questions portraying comparisons such as between interventions, programs, or approaches seem to be the most suitable to scoping studies. We also suggest assessing the quality of studies and conducting a trial of the method before fully embarking on the charting process in order to ensure consistency. The benefits of engaging a large, inter-professional team such as ours throughout every stage of Arksey and O’Malley’s framework far exceed the challenges and we recommend researchers consider the value of such a team. The strengths include breadth and depth of knowledge each team member brings to the study and time efficiencies. In our experience, the most significant challenges presented to our team were those related to consensus and resource limitations. Effective communication is key to the success of a large group. We propose that by clarifying the framework, the purposes of scoping studies are attainable and the definition is enriched.

## Background

Our research team, which is large and comprised of many disciplines, undertook a scoping study to investigate the information needs of people with colorectal cancer. We drew on the methods laid out in a 2005 framework by Arksey and O’Malley [1] who were among the first scholars to articulate a framework to clarify the usefulness of and methods inherent in a scoping study. They claim an interest in stimulating further discussion on the value of scoping studies and encourage others to further develop the methodology. Since their framework was published, a handful of researchers have taken up their challenge [2-8]. Levac et al. published the most prominent of these in 2010, addressing the framework’s strengths and limitations with the aim to encourage consistent methods across all scoping studies [8]. After reviewing our own research process and sharing our experiences with this methodology in this paper, we also contribute to the discussion that enhances Arksey and O’Malley’s framework. We offer a unique perspective on this framework because of our collective experience as a large, inter-professional team. We also propose that the definition, purposes, and methodological framework of scoping studies cannot be clarified in isolation but must be amended in tandem. We offer recommendations that, when added to those of other researchers such as Levac et al., generate a strengthened definition and position the purposes as attainable. Interestingly, some of our critiques and suggestions for the development of the methodology mirror those expressed by Levac et al. Since Levac et al’s paper was published while our team was concluding our scoping study; we reached our conclusions independently of their suggestions. The fact that we share some similar conclusions indicates that the collective suggestions from Levac’s team and ours are worth serious consideration for enhancing Arksey and O’Malley’s framework. We also offer some proposals for consideration not yet expressed by others engaged in scoping studies, primarily drawn from our experience as a large, inter-professional team. Our team of twelve people included academic researchers as well as clinical practitioners working in a cancer treatment centre from a range of disciplines and practices, including nutrition, radiation therapy, patient and family clinical counseling, nursing, biology, library and information science, and the social sciences. Each member of our team shared a common interest in colorectal cancer and a desire to improve the quality of care received by people with this diagnosis.

### What is a scoping study?

Over the last two decades, scoping studies have become an increasingly common method of searching the literature on a specific topic. There is no one agreed upon definition for scoping studies, but there has been some effort among researchers to seek clarification. Levac et al. confirm this view in their work, pointing to seven recent authoritative scoping sources, each offering a different definition or purpose. In spite of the varying interpretations in this methodology’s definition, and because we used the original definition as laid out by Arksey and O’Malley in our research, we refer to the Arksey and O’Malley definition throughout this paper.

Arskey and O’Malley begin with Mays et al’s definition of scoping studies: “to map *rapidly* the key concepts underpinning a research area and the main sources and types of evidence available, and can be undertaken as standalone projects in their own right, especially where an area is complex or has not been reviewed comprehensively before” [9]. The framework they developed serves to expand on this definition by identifying four main reasons for conducting a scoping study: (1) to examine the extent, range and nature of research activity; (2) to determine the value of undertaking a full systematic review; (3) to summarise and disseminate research findings; and (4) to identify research gaps in the existing literature. We identified the first and fourth goals as the most relevant for our own research. Arksey and O’Malley’s framework includes six stages, the sixth being optional: (1) identifying the research question, which is generally broad in nature; (2) identifying relevant studies, a process that is as comprehensive as possible; (3) study selection, with the establishment of inclusion/exclusion criteria, based on familiarity with the literature; (4) charting the data, a stage that includes sifting, charting, and sorting information according to key issues and themes; (5) collating, summarizing, and reporting the results, which provides both a descriptive and numerical summary of the data and a thematic analysis; and (6) a consultation exercise, an additional, parallel step involving key stakeholders to inform and validate study findings [1]. While Arksey and O’Malley’s framework offer the best framework for a scoping study to date, we argue that there is room for enhancement. Additional file 1 outlines the original framework, Levac et al’s recommendations, and our recommendations.

## Discussion

### Matching research interests with Arksey and O’Malley’s framework

Based on our understanding of Arksey and O’Malley’s framework, our research team believed a scoping study was a methodology that would work well to answer our research question: *What does the literature tell us about the information provided by health care professionals and needed by people with colorectal cancers across the cancer care continuum?* We applied to and received funding from a Canadian federal research granting institute under a program specific for scoping studies. Receipt of this funding endorsed our belief that this methodology would enable us to answer our question. However, as we engaged more deeply with our work using Arksey and O’Malley’s framework and came to understand our data, we developed reservations that we could claim to understand people’s information needs. When analyzing our final data set, we were tempted (or perhaps eager) to draw conclusions about our interest. Instead, we were faced with the dilemma: “what can we really say about this data?” It became clear that this methodology did not give us the right tools to extract the information we sought. We were able to identify what issues researchers *addressed*, but not *what information was wanted/needed by people with colorectal cancer*. In an effort to deal with this tension, we revised our research question mid-way through the process into a question that we hoped our collected data and the Arksey and O’Malley framework could answer: *What does the literature tell us about which aspects of the information provided by healthcare professionals and needed by people with colorectal cancer across the cancer care continuum have been addressed by researchers?*

Our goals in undertaking a scoping study were to examine the extent, range, and nature of research activity, and to identify research gaps in the existing literature as they relate to information needs of people with colorectal cancer. We quickly learned that our findings were insufficiently specific enough to allow us to fully meet our research goals *in the way we expected*. We were able to identify the gaps in what researchers were paying attention to with regard to information needs of people with colorectal cancer and we did understand the extent, range, and nature of research activity about the information needs of people with colorectal cancer – there had not been much research activity. However, even though we reached our goals, we were not able to answer our original question because we could not say anything *specific* about those needs. For example: What information do people with colorectal cancer say they want and/or need? How have these needs been articulated? How and when do they want these needs met? What is the reason given for these needs?

Nevertheless, these findings proved useful as they contributed to our understanding of the state of the literature and how we needed to proceed. Importantly, the study revealed to us that there is very little literature that draws on the perspective of people diagnosed with colorectal cancer. This inattention to people’s voice represented a gap in research. It became apparent that additional, in-depth research would be required before we could draw conclusions. Thus, our research has been an essential first step towards developing a research agenda where we truly attend to the information needs of people with colorectal cancer. Our findings provided us with enough data to design a further, more in-depth research project, thereby positioning the scoping study as an important initial step from which we launched the next phase of our research.

We recognise that we chose the methodology and perhaps it was not the best to answer our original question. We determined it was important to remain mindful of the types of conclusions we could reasonably draw from our data gathered through our use of Arksey and O’Malley’s framework. Thus, we believe that the conversation about the scoping study methodology that Arksey and O’Malley encourage could benefit from guidance about the kinds of research questions for which scoping studies are most appropriate. Arksey and O’Malley illustrate their framework describing a comparison between programs to support carers of people with mental health problems. A PUBMED search using the terms *scoping review* or *scoping study* not *systematic review* on July 27, 2012 elicited 101 articles published between July 2007 and July 2012; 45 of these articles describe studies that can be classified as scoping studies, according to Arksey and O’Malley’s definition, and 34 of them (75%) portray some type of comparison such as among interventions, programs, and approaches. It seems that this latter kind of research is one to which the scoping study method is most often applied and is perhaps the most useful. Our experience has led us to ponder the appropriateness of a scoping study to answer a question that is broader in nature than the comparison of interventions or programs. We were not interested in comparisons of interventions and programs, for example. Rather, we hoped to extract data that would tell us something concrete about the information needs of people with colorectal cancer. As a result, we propose that the objective and appropriateness of the methodology need to be clearly understood by those contemplating its use. However, the boundaries of this methodology and the types of research it can best support are still to be defined and we urge others to contribute to these definitions. It appears that this methodology may be best suitable to research that examines comparisons between interventions or approaches. On the other hand, we want to be clear that our research was not in vain as we did accomplish two goals laid out by Arksey and O'Malley and we garnered data that compelled us forward into a more in-depth analysis to answer our initial question in specific detail. Additionally, we were able to say useful things about the state of academic interest in issues relating to the information needs of people with colorectal cancer, the results of which have been already published [10]. Because our work proved useful in unexpected ways, we encourage others to consider a scoping review as a constructive method in their broader research process in that it can contribute to the clarification of next steps. By sharing our experiences with this methodology in this paper, we hope we can contribute to the enhancement of Arksey and O’Malley’s framework.

Our recommendations in this paper are the results of the review of our own research process. We collected our team’s perspectives through a short survey comprised of three open-ended questions: (1) describe any significant challenges/successes that arose from using the Arksey O’Malley framework for scoping reviews; (2) describe any significant challenges/successes that arose from taking part in research with a large, multi-disciplinary group; and (3) additional comments. Each team member’s response was built not only upon memory in hindsight, but also upon notes taken throughout the research process. At least two key team members kept research diaries and a recorder kept minutes of each bi-weekly meeting about the challenges, discussions, reflections, and actions taken. The survey results, the notes, and minutes were collated and analyzed. Analysis included identifying themes among challenges and successes, identifying relations between statements related to any given theme, stating arguments and supporting them with evidence from the materials, making comparisons, and evaluating conclusions. Results were then linked to specific Arksey and O’Malley methodological stages for reporting in this paper.

### Enhancing the six steps of Arksey and O’Malley’s framework: contributing to the conversation

#### Arksey and O’Malley’s first step: identifying the research question

Our research team initially found identifying an appropriate research question a straightforward task. We generated our question on the experience, observations, and concerns of our team’s health care providers and the findings of previous research projects of several team members. For example, after a diagnosis of colorectal cancer, there are multiple instances where information is exchanged and required by people diagnosed with colorectal cancer, their families, and health care providers. These may include diagnostic procedures, receiving the diagnosis, treatments and their side effects, and living beyond treatment. Our intent was to learn what information people with colorectal cancer needed and/or wanted, who or how it was best to provide it to them, and when. As per the framework’s first and fourth purposes for conducting a scoping study, we intended to examine the extent, range, and nature of research that addresses the information needs of people with colorectal cancer with a view to identifying gaps in the literature. With these two goals in mind, we linked our purpose to our research question. In an effort to advance the Arksey and O’Malley’s framework, Levac et al. suggest the need to clarify and link the purpose and the research question. Our team did so from the start.

In keeping with Arksey and O’Malley’s recommendation to avoid leading with a “highly focused research question,” [1] we believed our question was sufficiently broad in design; it helped us to capture an initial 10,753 papers. However, once our team delved into the literature and began analyzing the data, it became apparent that the research question required revision. Levac et al. propose researchers define the concepts in their research question to clarify the scope of the study. Similarly, Arksey and O’Malley suggest that “researchers will want to redefine search terms and undertake more sensitive searches of the literature” [1]. We held fast to the need for a question that was broad in nature. Levac et al’s useful advice was published after we finished collecting our data and had started our analysis. By this point in our research, we realized we could not say anything substantive about our research query. We revised our question to fit the data we collected instead of revising our question and literature search to get different data that would really illicit the specifics of our interest. In hindsight, we see Levac et al’s suggestion as complementary to Arksey and O’Malley’s suggestion.

To help us with this dilemma (and independent of Levac’s suggestion), we re-reviewed the 239 papers our study produced with an additional inclusion criteria – the voice of people with a diagnosis of colorectal cancer – and identified 64 articles that spoke to the information needs of people with colorectal cancer *from their point of view.* The realization of the need to clarify this research concept came late in our research process. Thus, it is only in retrospect that we realized how useful it would have been to us earlier. Had we added these inclusion criteria at the outset, we might have had fewer articles to analyse but they would have yielded the data we were looking for. We recommend that researchers follow this advice of Arksey and O’Malley and Levac et al. to reduce the need to adjust their question to fit the methodology (Additional file 1).

It is important to reiterate, however, that in spite of our challenging experience with Arksey and O’Malley’s framework, our scoping study was a productive research exercise. We learned what has captured the attention of researchers and by default, what has not. That we narrowed down the original 10,753 papers to a specific set of 239 papers indicates the limited amount of literature that addresses our subject [10]. Given the prevalence of colorectal cancer, we find this not only startling, but useful in directing our future research. We are now in the process of further examining the 64 papers that reveal the perspective of people with colorectal cancer.

#### Arksey and O’Malley’s second step: identifying relevant studies

This step requires comprehensiveness to be thorough. We searched for relevant studies using a general internet Google search and several electronic databases, including Google Scholar. In addition, we looked for current and closed clinical trials, practice guidelines, meeting abstracts and dissertations, and searched other sources of grey literature using the Grey Matters Checklist created by the 2011 Canadian Agency for Drugs and Technology in Health. Once we completed these electronic searches, we conducted a hand-search of four key journals and scanned the reference lists from relevant papers to identify other papers that may not have been found in the initial search. We also searched the Social Science Citation Index for papers that our initial searches may have missed [10]. We followed Arksey and O’Malley’s recommendation to conduct a very broad search since comprehensiveness is “the whole point of scoping the field” [1].

We started with a broad search strategy. Navigating and redefining the findings after this initial search added another level of complexity to the scoping process. “The process is not linear but iterative,” Arksey and O’Malley emphasize, “requiring researchers to engage with each stage in a reflexive way and, where necessary, repeat steps to ensure that the literature is covered in a comprehensive way” [1]. Specifically, Arksey and O’Malley suggest that researchers fine tune their search of the literature. As noted above, we neglected to do this step and, in hindsight, realize how useful it would have been. We agree that flexibility and comprehensive searches are important to successful scoping studies.

However, a fine balance must be struck between the laborious nature of study identification and the need for comprehensiveness on the one hand, with the need to complete a scoping study in a timely fashion, on the other. Levac et al. and others recognize this issue [2,4]. While Levac et al. recommend assembling a “suitable team with content and methodological expertise” [8] to identify studies, we recommend assembling a *small* suitable team to perform this task (Additional file 1). We were a large team and we quickly found it counterproductive for every team member to be actively involved at the beginning of the research process; with too many people, there may be the introduction of too many interpretations, thereby leading to inconsistent search results. In order to maintain consistency and control, we tasked our librarian team member with the design and execution of search strategies. Navigating this many references took considerable time and skill. Although our search produced such a significant number of references, it left our research team confident that we had scoped the field as comprehensively as possible. Additionally, the use of an online citation management software proved to be of immeasurable benefit. It allowed us to organize and cross-check our data and remove duplicates. It also allowed all those involved in the charting steps of the study easy access to the papers.

#### Arksey and O’Malley’s third step: study selection

Once we identified the relevant literature, our team established the exclusion and inclusion criteria to apply to the papers. The librarian and research assistant on our team applied these criteria to the paper abstracts. We included studies that mentioned in any way the information needs of people with a diagnosis of colorectal cancer, their families and caregivers, or the information sources used by them. We carefully set parameters around studies for exclusion. For example, we excluded studies related to screening, prevention, genetics, and studies that included other types of cancer in addition to colorectal cancer. By applying the exclusion criteria to the abstracts, we reduced the numbers of papers that needed to be read in detail to 869 papers.

Given our large team, we made an adjustment to Levac et al’s suggestion of engaging two reviewers to apply the inclusion/exclusion criteria to the papers. Specifically, we took a three-tiered approach to narrow down and cross-check our papers. First, we divided the research team into six teams of two people each. Each team was assigned one sixth of the 869 articles to review. Both members of each team independently reviewed the full text of each article to determine its eligibility for our study according to the inclusion and exclusion criteria. Second, team members then compared their results to reach an agreement about which articles they recommended to include or exclude. Third, if they could not reach an agreement, the research assistant or the librarian acted as a third reviewer. After concluding this process, a total of 239 papers met the inclusion criteria and were included in our study. We made this adjustment to Arksey and O’Malley’s framework due to the size of our research team. We wanted to give each of our twelve team members exposure to the data and to reduce the workload by dividing up the work tasks. Levac et al. reached a similar conclusion about how to adjust the application of the inclusion/exclusion criteria, confirming our own decision to modify this portion of the framework (Additional file 1).

Levac et al. and others [2,3,5] are concerned about Arksey and O’Malley’s framework’s inability to provide for an assessment of the quality of the literature. Because our research team is using our scoping study as the basis for our next stage of research, we did not focus too much on this concern (although it did concern us from the outset). However, in retrospect, we believe assessing for quality is a necessary component of scoping studies if they are to provide research that in itself can be disseminated to others in a way that is useful to practice or policymaking and for future researchers. We strongly recommend that assessing for quality be factored into Arksey and O’Malley’s framework and suggest adding these criteria to the selection of studies to be charted. Some suggested methods for assessing quality might include, for example, using validated instruments*.*

#### Arksey and O’Malley’s fourth step: charting the data

To organize data, Arksey and O’Malley recommend charting and sorting data according to key themes and issues. They suggest that data charted should include a mixture of general information about the study and specific information related to the study question. Arksey and O’Malley’s own scoping work, an examination of the literature about services or programs for a particular population, charted seven pieces of information on each article (general information: citation data, methodology, aims of the study, outcome measures, important results; specific information: intervention type, study population). Researchers can determine what categories to chart for their particular study. We opted to adapt a charting format from Rutten et al. [11], a systematic review investigating the information needs of people with cancer. The categories and subcategories used by Rutten et al. fit well with our research question as our goal was similar but focused on a specific type of cancer, colorectal. Because our team consisted of health care providers with extensive clinical experience, we were able to anticipate the complexity of patient information needs prior to conducting our scoping study. To this end, we expanded and modified the set of subcategories related specifically to information needs from the original 64 (from Rutten et al.) to a total of 82. Using such a comprehensive chart proved both a strength and a limitation of our research. It was a strength because our final set of data was rich. We collected more data than we had room to report in a single manuscript. On the other hand, it was time consuming to chart with such a large number of categories. Some of our team members who were busy, practicing health care providers found the detailed nature of the charting process challenging because it took time, first, to engage with the information we aimed to capture and, second, to actually capture that information.

Levac et al. suggest that research team members, independent of one another, chart the “first five to ten studies using the data-charting form and meet to determine whether their approach to data extraction is consistent with the research question and purpose” [8]. We undertook a similar step in that we conducted a trial charting exercise followed by a group consultation. This step added value by providing our team an opportunity to reach consensus about our data collection process and to include additional categories relevant to our research inquiry. It improved the quality and applicability of the chart used as well as consistency. We recommend researchers consider this step to ensure the data they collect are as rich as possible.

Using our revised Rutten chart, we repeated a similar process as in step three with our six teams of two members each, where each team member worked independently to chart the included articles and team members then compared their results to agree upon the charting of each article. However, we changed the membership of each team in an effort to ensure consistency in interpretation and validity in our results. The research assistant read and charted all 239 articles. All three reviewers discussed any discrepancies and agreed upon a final interpretation. Having one person read and chart each article ensured inter-reviewer reliability throughout the process and contributed to our confidence in the consistency of our data results; this process was an important and valuable part of our research. During this process, effective communication to maintain a clear framework and consistent charting methods cannot be underestimated, particularly for a large team.

Arksey and O’Malley’s framework recommended using Excel to chart each paper, but they did not advise their readers about a key feature of data management: the importance of assigning each paper a unique identifying number. We did this task early on as a way to conveniently and clearly track those articles we excluded and those we included. We also recommend this step because it provides a useful way for a large team to talk about a particular article without having to cite full references or get confused by the names of researchers who have published multiple papers.

It was during the charting step that we began to realize our inability to answer our research question. We charted that an article mentioned a particular information need, yet we could not say anything about that need. For example, did people with colorectal cancer say they wanted this information or did the authors note it as information given to people with colorectal cancer, and it was unconnected to the people receiving it?

#### Arksey and O’Malley’s fifth step: collating, summarizing, and reporting the results

When reporting the data in our study, it was imperative that we used a consistent, clear approach. Following Arksey and O’Malley’s framework, we charted the extent, nature, and distribution of the included articles. As previously mentioned, we made additions to the Rutten et al. charting categories to better suit the complex information needs of people with colorectal cancer. Our goal with the 15 categories (ten broadly defined categories of information needs and five broad information sources categories) was to gather high-level data about the types of information needs, while we hoped the 101 sub-categories (82 sub-categories of information needs and 19 sub-categories of information sources) would gather micro-level, specific data on these needs. We searched for this range of data in order to satisfy the varied interests of each of our inter-professional team members. These team members desired this level of detail in order to make sense of the data in relation to their own professional practice. This proved to be a real strength to our approach to scoping studies and affirmed the value of including health care providers on our research team. Our ability to capture a complexity of data did not necessarily get us closer to answering our original research question, but it did provide us with an indication of the types of information needs being discussed by researchers.

We made sense of our complex data with a focus on our research question. Once we gathered all of our data and produced some preliminary graphs portraying number of mentions and specific needs, we established a smaller working group to make meaning out of the data and to make choices about the data on which we should focus. By this point, we knew we were not answering our initial question. We then modified our question and followed Arksey and O’Malley’s suggestion to prioritize certain aspects of the literature according to implications for future research and what was most notable given our experience. With our research question in mind, we first quantified the data and produced graphs and charts to represent these numbers. We then did a thematic analysis that resulted in organizing our data into three overarching themes: the information needs of people with colorectal cancer, the sources of this information, and the timing of these information needs. Our steps mirrored Levac et al’s recommendation to add a stage resembling qualitative data analytical techniques, or a thematic analysis. We endorse Levac et al’s suggestion (Additional file 1).

We published our findings using a combination of tables with descriptions according to our themes [10]. This approach proved useful to us in our analysis and allowed us to clearly link our findings with our research goals: to examine the extent of the literature and to identify gaps. Our data also pointed very clearly to the need for future research to pay attention to the need for information as articulated by people with colorectal cancer. We do not offer a new recommendation in this regard, but support Levac et al. in their suggestions for this stage of the scoping study framework.

#### Arksey O’Malley sixth step: optional stage, consultation exercise

Arksey and O’Malley suggest an optional consultation stage with stakeholders, whereas Levac et al. suggest it should be a requirement. Based on our experience, we agree with Levac et al. (Additional file 1) but with room for interpretation of how that consultation is achieved. In our study, there were two groups of stakeholders: health care providers providing care to people with colorectal cancer and people with colorectal cancer themselves. Our research team included a significant number of health care providers. Their participation throughout the entire research process served the purpose of constant consultation and was a real strength of our inter-professional approach. We were able to extract multi-faceted perspectives in the literature and our findings *throughout* the entire research process rather than leaving it to the end of the project. As a result, we were able to revise the charting categories early on so it more accurately reflected people’s experience than if we had just borrowed one. The health care providers drew upon their experience to identify the gaps in the categories being un/attended to by researchers. We originally intended to consult with the second group of stakeholders, people diagnosed with cancer. However, we omitted that step after realizing that our study did not tell us anything about their information needs from their perspective because it gave us nothing substantive on which to seek their consultation. With Arksey and O’Malley’s framework, consultation only works if the results are germane to the group that researchers wish to consult. Moving forward with future research, we have built this consultation process into our current research with the 64 articles. We see it as a fundamental step to determine how what the literature says resonates with people with colorectal cancer.

Levac et al. and Brien et al. [5,8] discuss the importance of knowledge translation with stakeholders in the field, a perspective we share. In fact, Grant and Booth state that “scoping reviews are able to inform policymakers as to whether a full systematic review is needed” [4]. Although the methodology did not enable us to address our specific research question, the scoping study exercise did demonstrate that a further study is needed and it pointed out gaps in the literature. In fact, our experience led us to believe that scoping studies may be a useful step – perhaps even a necessary pre-requisite – toward a research project that allows for a deeper analysis, such as a knowledge synthesis methodology [12]. Indeed, our study has been valuable in part because it opened up questions to guide future research from which we will have findings we can share with stakeholders, including policymakers and health care providers to inform their practice.

Inviting suitable stakeholders to be part of the research team is one way to incorporate the consultation Levac et al. say is necessary. We recommend researchers consider this decision when possible as it makes the entire research process and, thus, the findings, rich. That being said, not all stakeholders will be appropriate to be involved on the research team and researchers must make this decision carefully. For example, previous experience with research is not necessarily a requirement but experience in a critical analysis of relevant literature is necessary. Effective team work skills are imperative, especially when large groups are assembled. Time availability to participate in all stages of the project is another important point to be considered.

### A large, inter-professional team using Arksey and O’Malley’s framework: strengths and challenges

Our contribution to the conversation on scoping studies relates to our experience as a large, inter-professional team using Arksey and O’Malley’s framework. Levac et al. and others point to a *multidisciplinary* team as a benefit for scoping studies [2,13]. According to Anderson et al., a multidisciplinary team provides the required “expertise to map a subject” that is “not necessarily always found in one researcher” [2]. Our research team was not only multidisciplinary, but inter-professional in its composition. A multidisciplinary team is academic in character because it joins together members trained in different disciplines, whereas an inter-professional team is professional in character because it joins together members trained in program areas of clinical practice, plus academic researchers [14]. It is important to note that the challenges we experienced are not unique to scoping studies, but are faced commonly by all research projects conducted by large, inter-professional teams. However, critical discussions about inter-professional teams are absent from the current literature about scoping study methods.

The large, inter-professional character of our team brought both strengths and challenges to Arksey and O’Malley’s framework. On one hand, it was a strength because our team collectively brought a breadth and depth of clinical and research knowledge absent in a team comprised only of academic researchers. It also brought time efficiencies. Our team worked efficiently when it came to assigning and completing work tasks. We strategically divided up work tasks, which often meant a lighter workload for each individual team member. This type of time efficiency was especially evident at the inclusion/exclusion and charting stages of Arksey and O’Malley’s framework. The make up of our team also added to the rigor of the research process; twelve team members from diverse backgrounds provided many perspectives.

On the other hand, the large, inter-professional character of our team had some challenges relating to consensus, research experience, and resource limitations. The greater the number of team members, the greater the number of perspectives. At certain points, productivity decreased. Although such a team produced rich analysis, it was challenging to reach consensus because there were occasional competing perspectives. At times, we found it difficult to arrive at a common language or vocabulary for discussion and had to re-double our efforts during the research process to ensure consistency.

Finally, as with any research project, there is always a need to balance finite resources of time and money. With an inter-professional team, this need is greater because there are more competing interests, more professional schedules to coordinate, and more communication efforts required to explain, explore, and reach consensus about ideas. We found it helpful to designate one person or a small section of the team to take the lead at various stages of the research process to move the project forward. While we have noted at several steps of the process that communication and regular meetings are important, we also acknowledge that it may be challenging to organize meetings for so many team members and to ensure that all team members consistently produce deliverables at agreed upon deadlines. We recommend, however, that large, inter-professional teams pay special attention to this issue as communication is key to success.

## Summary: lessons learned

Some authors have expressed their concerns about Arksey and O’Malley’s framework’s inability to provide for an assessment of the quality of the literature [2,3,5,8]. We believe assessing for quality is a necessary component of scoping studies. The assessment itself is a significant task and should be performed using validated instruments. This recommendation does not stand alone, however. We propose that the recommendations of Levac et al. to clarify the concepts in the research question and Arksey and O’Malley’s suggestion to redefine search terms will ensure the assessment of quality actually proves useful. To this end, the definition and purposes of scoping studies must be made in tandem with clarifying the methodological framework; no one can be done without the others.

We endorse Arksey and O’Malley’s purposes when taking into consideration Levac et al. and our own recommendations. As for the definition used by Arksey and O’Malley, we would remove the term “rapidly” and replace it with the need for scoping studies to be done thoroughly and thoughtfully. To be clear, scoping studies take time. Second, we think that adding a quality assessment step would alter the definition.

With a more detailed methodological framework according to the recommendations of Levac et al., our own, plus purposes that are more attainable, we suggest a definition of scoping studies as follows: *Scoping studies aim to map the literature on a particular topic or research area and provide an opportunity to identify key concepts; gaps in the research; and types and sources of evidence to inform practice, policymaking, and research*.

Creating a large, inter-professional team to engage in a scoping study offers tangible benefits to the research process. If using a revised framework set out by Arksey and O’Malley’s using Levac et al. and our recommendations, major tasks can be fairly divided without compromising the consistency of analysis. Additionally, each inter-disciplinary team member adds richness to the analysis as he/she brings his/her perspective to the research table.

A large, inter-professional team including health care providers allowed us to anticipate the complexity of information needs. Including suitable stakeholders on the research team builds in the consultation stage throughout and adds depth to all stages of the research process.

It is important that researchers fully understand the methodology’s boundaries and its inherent methods. It would be beneficial to include someone on the research team who is experienced with scoping studies. Most important is the work involved of matching the methodology with research interests and attending to suggestions to clarify concepts within the research question and/or focus the literature search as necessary to ensure researchers do not lose sight of their interests.

## Competing interests

All authors declare that they have no competing interest.

## Authors’ contributions

Each author (HMLD, CvM, and SJS) made substantial contributions to the conception, analysis, and interpretation of the data. Following a group discussion about the outline of the paper, SJS created the first draft of the manuscript, HMLD and CvM revised the draft and, thereafter, each author interactively participated in critically revising the content up to the final, submitted version. Each author read and approved the final manuscript.

## Pre-publication history

The pre-publication history for this paper can be accessed here:

http://www.biomedcentral.com/1471-2288/13/48/prepub

## Supplementary Material

Additional file 1: Table S1 Comparative recommendations to enhance Arksey and O’Malley’s six framework stages. Click here for file
