# Supplementary material for: Enhancing the scoping study methodology: a large, inter-professional team’s experience with Arksey and O’Malley’s framework
Source: BMC Med Res Methodol. 2013 Mar 23;13:48. doi: 10.1186/1471-2288-13-48 (PMC3614526; doi:10.1186/1471-2288-13-48)
Supplement: Additional file 1: Table S1 — Comparative recommendations to enhance Arksey and O’Malley’s six framework stages. [file 1471-2288-13-48-S1.doc]

| Table S1Comparative Recommendations to Enhance Arksey and O’Malley’s Six Framework Stages | | |
| --- | --- | --- |
| Arksey and O’Malley’s Framework Stage(2005) | Levac et al’s Recommendations(2010) | Our Recommendations(2012) |
| #1 Identifying the research question: This step guides the search strategy and all the subsequent steps. Research questions should be broad in nature in order to generate breath of coverage. | 1. Clearly articulate the research question that will guide the scope of inquiry. Consider the concept, target population, and health outcomes of interest to clarify the focus of the scoping study and establish an effective search strategy.  2. Mutually consider the purpose of the scoping study with the research question. Envision the intended outcome (*e.g*., framework, list of recommendations) to help determine the purpose of the study.  3. Consider rationale for conducting the scoping study to help clarify the purpose. | 1. Conduct considerable research about scoping studies to ensure an appropriate match between the scoping methodology and the research interest. Consider the methodology’s objectives, boundaries, and the types of research that it can best support.  2. Link the purpose of the research with the research question and attend to suggestions to clarify concepts within the research question. |
| #2 Identifying relevant studies: This step is as comprehensive as possible and involves searching for research evidence using different sources | 1a. Research question and purpose should guide decision-making around the scope of the study.  1b. Assemble a suitable team with content and methodological expertise that will ensure successful completion of the study.  1c. When limiting scope is unavoidable, justify decisions and acknowledge the potential limitations to the study. | 1. Remain flexible to revise the research question and/or search terms.  2. Build both a multidisciplinary and inter-professional team. Include someone experienced with scoping studies and suitable stakeholders if possible.  3. Choose a small suitable group from the larger research team of qualified researchers and professionals with enough breadth of expertise for this stage to ensure timely completion of the study. |
| #3 Study selection: This step is based on inclusion/exclusion criteria developed post hoc after familiarity with the literature is established. A team approach is suggested but not imperative. | 1. This stage should be considered an iterative process involving searching the literature, refining the search strategy, and reviewing articles for study inclusion.  2a. At the beginning of the process, the team should meet to discuss decisions surrounding study inclusion and exclusion. At least two reviewers should independently review abstracts for inclusion.  2b. Reviewers should meet at the beginning, midpoint and final stages of the abstract review process to discuss challenges and uncertainties related to study selection and to go back and refine the search strategy if needed.  2c. Two researchers should independently review full articles for inclusion.  2d. When disagreements on study inclusion occur, a third reviewer can determine final inclusion. | 1. For large research teams, take a three-tiered approach to study selection. Divide entire team into smaller teams with responsibility for equal portions of the selected studies. Ask each person to review his/her selected studies for inclusion or exclusion. Have each small team compare its results. If disagreement, involve a third reviewer.  2. Assess the quality of studies to be either included or excluded for charting. Quality can be assessed using validated instruments |
| #4 Charting the data: This step consists of collecting data according to key issues and themes. Two main categories of data are suggested: general information about the study and specific information related to the research question. | 1a. The research team should collectively develop the data-charting form and determine which variables to extract in order to answer the research question.  1b. Charting should be considered an iterative process in which researchers continually extract data and update the data-charting form.  1c. Two authors should independently extract data from the first five to ten included studies using the data-charting form and meet to determine whether their approach to data extraction is consistent with the research question and purpose.  2. Process-oriented data may require extra planning for analysis. A qualitative content analysis approach is suggested. | 1. Conduct a trial charting exercise and group consultation to determine if adjustments should be made to the chart (variables being measured) and to ensure that the research team is charting consistently.  2. Create a comprehensive chart, involving both high-level data and micro-level data, in order to capture a rich set of data.  3. Hold frequent meetings to ensure effective communication about consistent charting. Hold additional longer meetings when necessary.  4. For large research teams, take a three-tiered approach to charting the data. Divide entire team into smaller teams with responsibility for equal portions of the selected studies. Pick different team members from stage #3. Ask each person to review his/her selected studies for inclusion or exclusion. Have each small team compare its results. Have one independent reviewer read and chart all studies. Have independent reviewer compare his/her charting with the charting of all other team members. Discuss any discrepancies.  5. Improve data management by assigning each study a unique identifying number to avoid confusion. |
| #5 Collating, summarizing, and reporting the results: This step includes a descriptive numerical summary related to the general information collected and a thematic construction of the specific information collected. | Researchers should break this stage into three distinct steps:  1a. Analysis (including descriptive numerical summary analysis and qualitative thematic analysis);  1b. Reporting the results and producing the outcome that refers to the overall purpose or research question;  1c. Consider the meaning of the findings as they relate to the overall study purpose; discuss implications for future research, practice and policy. | 1. Engage a small working group from the larger team to make meaning out of the data and to make choices about the data on which to focus. |
| #6 Consultation; This step is optional. Consultation with key stakeholders may provide additional sources of information and offer different perspectives on the data collected. | 1. Consultation should be an essential component of scoping study methodology.  2a. Clearly establish a purpose for the consultation.  2b. Preliminary findings can be used as a foundation to inform the consultation.  2c. Clearly articulate the type of stakeholders to consult and how data will be collected, analyzed, reported and integrated within the overall study outcome.  2d. Incorporate opportunities for knowledge transfer and exchange with stakeholders in the field. | 1. If there are stakeholders who were not part of your research team, engage in a consultation process with them. Consult stakeholders only if the actual scoping study results are germane.  2. Recognize that the inability to share a scoping study’s findings with stakeholders may be an indication that future research must be done beyond the scoping study in order to make a meaningful contribution to professional practice. |
